# Editorial Hint on Grafting

**Published:** 1856-02

**Authors:** 


### EDITORIAL HINT ON GRAFTING.

We suggest to Editors who have a wish to elevate the "Profession" rather than tumble it down to the bottom of the ladder with sawyers, boot blacks, patent medicine makers, and the like, to make it a practice to read carefully, every article they think of putting in their paper; they will often save their reputation by it, and at the same time they will avoid the perpetration of many precepts about health whose absurdity a moment's reflection would detect. A statement has been going through the papers for a month past as "An interesting discovery from *France*," that the best mode of engrafting fruit trees is to stick one end of a "slip" in a potatoe the other end projecting two or three inches. We certainly saw this method practiced in the West when we were a baby, for soon after that we did it ourselves, understanding the reason to be that the potatoe gave moisture long enough to make the roots strike out.

